# Immunization Strategies in Pediatric Patients Receiving Hematopoietic Cell Transplantation (HCT) and Chimeric Antigen Receptor T-Cell (CAR-T) Therapy: Challenges and Insights from a Narrative Review

**DOI:** 10.3390/vaccines13090932

**Published:** 2025-09-01

**Authors:** Daniele Zama, Laura Pedretti, Gaia Capoferri, Roberta Forestiero, Marcello Lanari, Susanna Esposito

**Affiliations:** 1 Pediatric Emergency Unit, IRCCS, Azienda Ospedaliera Universitaria di Bologna, 40138 Bologna, Italy; daniele.zama2@unibo.it (D.Z.); marcello.lanari@unibo.it (M.L.); 2 Pediatric Clinic, Department of Medicine and Surgery, University of Parma, 43126 Parma, Italy; laurapedre@hotmail.it (L.P.); gaia.capoferri@unipr.it (G.C.); roberta.forestiero@unipr.it (R.F.)

**Keywords:** CAR-T therapy, hematopoietic cell transplantation, immunization strategies, immunocompromised children, pediatric infectious diseases, vaccine-preventable diseases

## Abstract

Background: Hematopoietic cell transplantation (HCT) and chimeric antigen receptor T-cell (CAR-T) therapy have markedly improved survival in pediatric patients with hematological malignancies. However, these treatments cause profound immunosuppression, leading to significant susceptibility to vaccine-preventable diseases (VPDs), including invasive pneumococcal disease and measles. Timely and tailored immunization strategies are crucial to mitigate infectious risks in this vulnerable population. Methods: We conducted a narrative review of the English-language literature from 2000 to 2024, including clinical guidelines, surveys, and original studies, to evaluate immune reconstitution and vaccination practices in pediatric patients undergoing HCT and CAR-T therapy. Literature searches in PubMed, Scopus, and Web of Science used disease-specific, therapy-specific, and pathogen-specific terms. Data synthesis focused on vaccine schedules, immune recovery markers, and adherence challenges. Results: Profound immune deficits post-HCT and CAR-T therapy compromise both innate and adaptive immunity, often necessitating revaccination. Key factors influencing vaccine responses include time since therapy, graft source, immunosuppressive treatments, and chronic graft-versus-host disease. Although inactivated vaccines are generally safe from three to six months post-HCT, live vaccines remain contraindicated until documented immune recovery. CAR-T therapy introduces unique challenges due to prolonged B-cell aplasia and hypogammaglobulinemia, leading to delayed or reduced vaccine responses. Despite established guidelines, real-world adherence to vaccination schedules remains suboptimal, driven by institutional, logistic, and patient-related barriers. Conclusions: Effective vaccination strategies are essential for reducing infectious morbidity in pediatric HCT and CAR-T recipients. Personalized vaccine schedules, immune monitoring, and multidisciplinary coordination are critical to bridging gaps between guidelines and practice, ultimately improving long-term outcomes for immunocompromised children.

## 1. Introduction

Advances in immunotherapy, including hematopoietic cell transplantation (HCT) and chimeric antigen receptor T-cell (CAR-T) therapy, have significantly improved outcomes for pediatric patients with cancer. However, these innovative treatments introduce complex challenges related to immune suppression and an increased risk of infections.

HCT remains a cornerstone intervention for a variety of malignant and non-malignant pediatric conditions [1]. Nevertheless, children undergoing HCT experience profound and prolonged immunosuppression, rendering them highly susceptible to infections. Critically, this vulnerability includes the loss of immunity previously acquired through natural infections and routine childhood vaccinations. Such immunological deficits stem from the intensive chemotherapy, radiation, and immunosuppressive regimens administered before, during, and after transplantation, which result in significant depletion of both the quantity and functionality of lymphocytes. Consequently, immunological memory established through prior vaccinations is often severely compromised, diminishing the host’s capacity to mount effective antibody and cell-mediated immune responses. Because conditioning regimens effectively “reset” the immune system, pediatric HCT recipients require comprehensive revaccination to restore protection against vaccine-preventable diseases (VPDs), including viral infections and those caused by encapsulated organisms such as *Streptococcus pneumoniae*, *Haemophilus influenzae* type b, and *Neisseria meningitidis* [2,3,4].

VPDs contribute substantially to morbidity and mortality during the early post-transplant period. A European multicenter survey demonstrated that the risk of invasive pneumococcal disease (IPD) is markedly higher in transplanted children—especially those with active graft-versus-host disease (GVHD) and recipients of allogeneic HCT—compared with autologous transplant recipients [3]. Moreover, late-onset infections, occurring more than 100 days post-HCT, are more prevalent than early IPD. A striking example of the consequences of inadequate immunity in this population is the measles outbreak reported by Ge et al. in 2015, involving pediatric patients with hematologic and oncologic conditions in Shanghai [4]. The outbreak exhibited a mortality rate exceeding 20%, underscoring the critical importance of timely measles vaccination and stringent infection control measures in high-risk clinical settings.

Current guidelines recommend initiating vaccinations between three and six months post-transplant, contingent upon sufficient immune reconstitution [5,6,7]. However, vaccination timing must be individualized, considering factors such as the presence of GVHD and ongoing immunosuppressive therapy. In such cases, inactivated vaccines may be delayed mainly to ensure adequate immune response, whereas live-attenuated vaccines are contraindicated until immune recovery is documented due to the risk of vaccine-related adverse events. During the early post-HCT period, non-live vaccines—including inactivated influenza, pneumococcal vaccines, hepatitis B, DTaP, IPV, Hib, meningococcal vaccines, and SARS-CoV-2 mRNA vaccines—are generally considered safe, as they do not replicate in the host. In contrast, live-attenuated vaccines (e.g., varicella, MMR, and recombinant zoster) remain contraindicated until there is unequivocal evidence of immune recovery. Other live vaccines—such as rotavirus, Bacillus Calmette–Guérin (BCG), and certain travel-related vaccines like yellow fever or Japanese encephalitis—also remain contraindicated in HCT recipients [5,6,7]. Table 1 summarizes recommended vaccines for children undergoing HCT.

CAR-T cell therapy represents a major breakthrough in the treatment of hematological malignancies, particularly in pediatric patients [8]. By engineering T lymphocytes to express chimeric receptors specific for tumor antigens, CAR-T therapy effectively harnesses the immune system to target and eliminate malignant cells. However, this therapy is also associated with profound immunosuppressive effects, predisposing patients to a significant risk of infections. As CAR-T therapy becomes more widely implemented, understanding its infectious complications and optimizing preventive strategies are essential [8].

In this narrative review, we focus on immune reconstitution as a critical aspect influencing vaccination strategies in pediatric patients undergoing HCT and CAR-T therapy. Key immune parameters—including T-cell counts and function, B-cell function (as reflected by immunoglobulin levels), and the abundance of antigen-presenting cells—are closely correlated with vaccine efficacy [1]. Additionally, we explore vaccine adherence and follow-up among transplant patients, their families, and healthcare workers, recognizing these as crucial factors in preventing infections in immunocompromised individuals.

## 2. Methods

This narrative review was conducted through a comprehensive literature search of peer-reviewed articles, clinical guidelines, and relevant publications focused on immunotherapy, HCT, CAR-T therapy, immune reconstitution, and vaccination strategies in pediatric oncology.

Systematic searches were performed in PubMed, Scopus, and Web of Science using combinations of relevant keywords, including terms related to immunization (e.g., “vaccination,” “vaccine,” “immunization,” “immune response,” “donor vaccination”) and pediatric-specific terms such as “pediatric,” “paediatric,” and “infant.” Searches also incorporated transplant-related terms (e.g., “bone marrow transplantation,” “hematopoietic stem cell transplantation,” “HCT,” “HSCT,” “peripheral blood stem cell transplantation,” “umbilical cord blood transplantation”), immunotherapy-related terms (e.g., “CAR-T,” “chimeric antigen receptor T-cell,” “CAR T-cell therapy,” “adoptive T-cell therapy”), and pathogen- or vaccine-specific terms such as “*Streptococcus pneumoniae*,” “*Haemophilus influenzae*,” “*Neisseria meningitidis*,” “tetanus,” “diphtheria,” “pertussis,” “influenza,” “hepatitis B,” “poliomyelitis,” “human papillomavirus,” “varicella zoster,” “measles,” “mumps,” “rubella,” “yellow fever,” “dengue,” “rotavirus,” “BCG,” “live-attenuated vaccines,” “monkeypox,” “SARS-CoV-2,” and “COVID-19.” These terms were combined in various configurations to maximize the breadth and relevance of search results.

Eligible publications included English-language articles published between 2000 and 2024, encompassing clinical guidelines, consensus statements, position papers, retrospective reviews, scientific surveys, and case reports. Special emphasis was placed on current vaccination recommendations for pediatric patients undergoing HCT and CAR-T therapy. Additional references were identified through manual screening of citations in key articles and reviews.

Identified studies were assessed for relevance, and findings were synthesized to provide an updated overview of immune-related complications and vaccination strategies in immunocompromised pediatric populations. Particular focus was given to studies addressing immune reconstitution following HCT, including comparisons between autologous and allogeneic transplantation to highlight differences in immune recovery dynamics. Furthermore, retrospective analyses were reviewed to underscore the importance of individualized revaccination strategies and continuous monitoring aimed at improving vaccine uptake and adherence in this vulnerable patient group.

In total, our literature search identified over 350 publications, of which 112 met our inclusion criteria and were ultimately incorporated into this review. Prioritization was based on the relevance of the study design and population: we included clinical guidelines, consensus statements, and systematic reviews as the primary sources for recommendations; large multicenter or national cohort studies were emphasized to support generalizable findings; and single-center experiences, case reports, or expert opinions were cited selectively when they provided unique insights or addressed gaps not covered by higher-level evidence. When conflicting recommendations were encountered, priority was given to the most recent consensus guidelines (i.e., European Conference on Infections in Leukaemia Center for Disease Control, Infectious Disease Society of America, American Society of Blood and Marrow Transplantation, and the European Society for Blood and Marrow Transplant) to ensure that the synthesis reflected current expert agreement.

## 3. Hematopoietic Stem Cell Transplantation in Pediatric Recipients

### 3.1. Immune Reconstitution

The timing of immune reconstitution after HCT varies considerably among patients and is influenced by multiple factors related to the recipient, donor, and transplant protocols. Individual characteristics and laboratory markers—including thymic function and hormonal levels—may help predict the immunologic response to revaccination [9]. Although hematopoietic engraftment and donor chimerism are typically achieved early, significant immune deficits often persist for months in the post-transplant period [10]. Several factors have been associated with suboptimal vaccine responses, including a shorter interval since transplantation, older recipient age, and recent administration of immunosuppressive therapies [11,12,13,14,15]. The choice of stem cell source also plays a critical role; for example, grafts derived from umbilical cord blood or CD34^+^-selected products offer limited passive transfer of donor-derived memory immune cells compared to unmanipulated bone marrow or peripheral blood stem cell sources [16,17,18].

Immune reconstitution has become a central focus in HCT due to the elevated risk of life-threatening opportunistic infections and the association between delayed immune recovery and disease relapse [19]. To mitigate these risks, intensive supportive measures—including antimicrobial prophylaxis and immunoglobulin replacement therapy—are frequently employed [19]. Innate immunity typically recovers earliest: neutrophil engraftment usually occurs around days 15–20 post-allogeneic HCT, defined as achieving ≥500 cells/μL for three consecutive days [18,19]. Granulocytes, monocytes, and dendritic cells normalize within weeks, and NK cells often reconstitute within 1–4 months, regardless of graft source. Although detailed data on NK subsets are limited, early NK recovery has been associated with lower infection risk and improved survival [18,19].

In contrast, recovery of adaptive immunity is significantly slower and more heterogeneous [18]. T-cell reconstitution is dependent on thymic output, making older recipient age and thymic injury key barriers. CD8^+^ cytotoxic T cells typically normalize within 9–12 months, whereas CD4^+^ helper T-cell recovery may take over a year, resulting in a prolonged inverted CD4/CD8 ratio [20]. Chronic GVHD and its therapies further impair CD4^+^ recovery, with thymic damage leading to prolonged deficits in naive T-cell production [20]. Monitoring of immune reconstitution in clinical practice is most commonly performed by flow cytometry, assessing CD19^+^ and CD20^+^ B cells, CD4^+^ and CD8^+^ T-cell subsets, as well as naive and memory populations, including circulating T follicular helper (cTfh) cells, which are critical for germinal center responses. T-cell receptor excision circles (TRECs) are also used to quantify thymopoiesis and naive T-cell output [20].

B-cell recovery is also delayed and depends on graft source, steroid exposure, and T-cell help. Importantly, anti-thymocyte globulin (ATG) primarily targets T cells and does not directly deplete B-cell precursors, although B-cell recovery may be secondarily impaired by the lack of T-cell support and by immunosuppressive therapy for GVHD. Memory B-cell subsets reconstitute slowly, often requiring up to two years to normalize. Switched memory B cells (CD19^+^CD27^+^IgD^−^IgM^−^) generally recover earlier than IgM memory B cells (CD19^+^CD27^+^IgD^+^IgM^+^) [18]. Chronic GVHD is associated with persistently low IgG- and IgA-producing B cells, compounding susceptibility to encapsulated bacterial infections [20].

Clinical outcomes of immune recovery differ by transplant type. Allogeneic HCT remains the predominant procedure worldwide and is associated with slower, more incomplete immune reconstitution compared to autologous HCT, due to the impact of conditioning intensity, graft-versus-host disease, and prolonged immunosuppression [17,18]. In contrast, autologous HCT recipients typically achieve faster B- and T-cell recovery, which translates into more reliable vaccine responses. Future improvements in immune recovery are expected to focus on alloHCT strategies, given their higher frequency and clinical relevance.

A prospective study at the Children’s Hospital in Helsinki followed 51 pediatric alloHCT recipients (mean age: 8 years) and illustrated these dynamics [20]. Recovery of CD3^+^, CD4^+^, and CD8^+^ T-cell counts and TRECs was delayed in patients with moderate to severe chronic GVHD. Early CMV and EBV reactivations were associated with impaired naive T-cell recovery and reduced thymic output. B-cell reconstitution (CD19^+^ and CD20^+^ counts) was also delayed in patients with GVHD, and early viral infections negatively impacted B-cell recovery, which remained incomplete in some children even one year post-HCT [20].

Two main approaches are currently used to determine the optimal timing for revaccination after HCT: time-based and immune reconstitution-based strategies. The traditional time-based approach, endorsed by many consensus guidelines, recommends restarting inactivated vaccines between 3 and 6 months post-transplant and live vaccines after at least 24 months, provided there is no GVHD or ongoing immunosuppression [5]. This method is straightforward, easily applicable in clinical practice, and facilitates compliance, particularly in centers with limited access to routine immune monitoring. In contrast, the immune milestone-based approach seeks to individualize vaccine timing by linking initiation to specific markers of immune recovery. Thresholds commonly cited include CD4^+^ T-cell counts > 400 cells/µL, serum IgG > 400 mg/dL, and an absolute lymphocyte count > 1000 cells/µL, as described by Haynes et al. [16] and further elaborated in studies by Forlenza and Small [21], Small et al. [22], and Ljungman [23]. This strategy aims to optimize vaccine immunogenicity and minimize the risk of inadequate serologic responses, though it requires frequent laboratory assessments and may be more resource-intensive. In practice, many centers adopt a hybrid model, using a time-based framework for feasibility while incorporating immune monitoring when available to refine the timing of revaccination.

A multicenter randomized trial in the United States assessed immune responses to high-dose trivalent versus standard-dose quadrivalent influenza vaccines in children aged 3–17 years, vaccinated between 3 and 35 months post-alloHCT [24]. Higher numbers of circulating CD4^+^ naive and memory T follicular helper (cTfh) cells were correlated with stronger and more durable vaccine responses. These findings underscore the critical role of cTfh cells in promoting B-cell function and suggest that detailed phenotyping of T- and B-cell subsets could inform personalized vaccine strategies in immunocompromised children [24].

Data from Wiegering et al. demonstrated that autologous HCT recipients generally experience faster immune recovery than allogeneic recipients, particularly in the restoration of naive T-cell subsets [18]. However, this more rapid recovery did not translate into a survival advantage, underscoring the complexity of immune reconstitution dynamics between different transplant types.

Some patients may retain protective antibody titers to selected pathogens after HCT, particularly following autologous procedures or in those with less profound immunosuppression [17,18,22,23,24,25,26]. This preserved seropositivity can provide interim protection and may influence the timing of certain boosters. However, persistence of antibody levels should not be equated with full immune reconstitution, which requires recovery of both cellular and humoral compartments to support long-term immunity and responses to new antigens [16,17]. Accordingly, serological testing may help guide individualized revaccination, but comprehensive assessment of immune recovery remains essential for decisions such as the introduction of live vaccines [16,17,18,22,23,24,25,26,27,28,29].

In a Turkish cohort, 173 pediatric HCT recipients completed inactivated and 131 completed live vaccines after meeting predefined immunologic criteria [26]. The median time to revaccination was 15 months, with seroprotection rates exceeding 90% for hepatitis A and B, pertussis, and measles. No significant differences in serologic responses were observed based on sex, graft source, chimerism status, IgG recovery time, or immunosuppressive regimens [26].

A retrospective study from Children’s Hospital Colorado reported high post-revaccination seroprotection rates of 100% for rubella, tetanus, and diphtheria, and 98% for Hib, while lower rates were observed for varicella (25%), mumps (67%), and measles (76%) [16]. Poor vaccine responses were associated with older age, exposure to rituximab, total body irradiation (TBI), and the use of cord blood grafts.

Similarly, in Helsinki, a protocol based on the timing of revaccination and immune recovery achieved high rates of seroprotection in 23 pediatric alloHCT survivors, with particularly robust responses to hepatitis B and pneumococcal vaccines [27].

A Turkish study comparing TCRαβ-depleted haploidentical HCT recipients to those receiving fully matched grafts found comparable seroprotection after a complete revaccination schedule initiated at 12 months, supporting the feasibility of applying standard vaccine protocols in haploidentical recipients with adequate immune recovery [30].

### 3.2. Vaccine Adherence and Follow-Up in Transplant Patients

Despite the availability of consensus guidelines for immunization in pediatric oncology and HCT settings, significant deviations from recommended practices persist in clinical practice. A single-center survey by Pettke et al. revealed suboptimal influenza vaccination rates among pediatric oncology patients, their families, and even healthcare workers, with adherence falling below 50% across all groups [31]. Using self-administered questionnaires, the study identified primary motivators for vaccination—including fear of transmitting infections, fear of illness, and recommendations from medical staff—as well as major barriers such as concerns over side effects, skepticism regarding vaccine efficacy, and a perceived low risk of infection [31].

In a quality improvement initiative conducted in Philadelphia, a multifaceted educational campaign targeting healthcare workers and patient families successfully increased adherence to annual influenza vaccination [32]. Emphasizing the importance of vaccination in protecting immunocompromised cancer patients, the intervention increased vaccination rates significantly, from 20.1% to 64.5% [32]. These findings highlight the crucial role of healthcare workers and family members in promoting vaccine uptake—not only through patient education but also by leading by example.

According to guidelines, individuals in close contact with HCT recipients should be fully vaccinated according to national immunization schedules, particularly for varicella, measles, mumps, and rubella [33]. Annual administration of the inactivated influenza vaccine is strongly recommended for family members and caregivers both before transplantation and during the immunocompromised period [33].

A persistent gap between evidence-based guidelines and real-world practice remains a significant obstacle to achieving effective vaccine coverage, as highlighted by several studies [34,35,36]. Vaccination schedules are often complex, involving multiple vaccines administered at varying intervals. Given the heightened risk of complications in oncology patients, meticulous assessment by specialized multidisciplinary teams is critical to determine optimal vaccine timing. Errors in this process may lead to delays or missed revaccination opportunities [1,37].

While individualized vaccination schedules based on immune reconstitution markers may optimize immunogenicity, it is important to acknowledge that routine time-based vaccine schedules have the distinct advantage of simplicity and feasibility. These protocols can be more readily implemented by primary care providers without the need for frequent immunological monitoring or specialist input, thereby avoiding the complexity of a multidisciplinary approach [1,37]. Importantly, the ease of application translates into higher adherence and improved vaccination coverage rates, ensuring that a greater proportion of HCT survivors receive recommended immunizations in a timely manner. This public health benefit underscores why time-based schedules remain widely adopted in many centers, even as immune milestone-based approaches gain increasing attention [1,37].

An essential component of post-HCT vaccination programs is the assessment of immune response to vaccination. Serological testing after selected vaccines (e.g., hepatitis B, tetanus, pneumococcus, measles, mumps, rubella, and varicella) provides useful information on the adequacy of protective immunity and can guide subsequent vaccine decisions [37,38]. For hepatitis B, antibody titers may be checked after completion of the primary vaccine series to confirm protection, and additional doses can be considered for non-responders, although routine revaccination schedules remain the primary strategy [33,34]. In contrast, for MMR, serology may help identify patients with preserved immunity, but because antibody persistence is often unreliable and cellular responses are critical, most guidelines recommend revaccination once immune reconstitution criteria are met, regardless of baseline serostatus [17,33,35]. Overall, documented seroconversion or protective antibody titers may support continuation of vaccine schedules, while absent or suboptimal responses can justify additional doses. Importantly, serology is also relevant to the timing of live-attenuated vaccines: some guidelines suggest that demonstration of protective antibody responses to inactivated vaccines can serve as a threshold marker of sufficient immune recovery to safely introduce live vaccines such as MMR or varicella, in addition to the standard time-based and immunological milestones [17,18,39]. Serology should therefore be viewed as a complementary tool, rather than a routine requirement for all vaccines, within structured revaccination protocols.

In the first year after HCT, patients often have impaired responses to a single influenza vaccine dose. To improve protection, many guidelines recommend administering two doses of inactivated influenza vaccine, given one month apart, during the first post-transplant influenza season [17,33,35]. This approach is supported by studies showing higher seroconversion rates with a two-dose schedule in immunocompromised hosts. Annual single-dose vaccination is then continued in subsequent years.

A cohort study from Brazil highlighted additional challenges to vaccine adherence among HCT recipients, particularly poor communication between transplant and vaccination centers and limited patient compliance [37]. Notably, institutional factors accounted for over 50% of revaccination delays, while patient non-adherence contributed to approximately 20% [37].

Vaccines requiring multiple doses or formulations pose further logistical challenges. Nelson et al. found low adherence to multidose vaccines (e.g., varicella, hepatitis A and B), particularly among adolescents, young adults, and individuals of lower socioeconomic status [38]. Beyond dosing complexity, missed vaccinations were often associated with patient attrition. A longitudinal U.S. study reported that pediatric HCT survivors aged 10–17 years were at a higher risk of being lost to follow-up than younger children, across both allogeneic and autologous transplant groups [39].

Clinical conditions such as GVHD and immunosuppressive therapy frequently necessitate reevaluation of vaccination timing [33]. However, ECIL-7 advises that only live-attenuated vaccines be deferred in cases of severe GVHD, active disease relapse, ongoing significant immunosuppression, or hypogammaglobulinemia (<3 g/L), while inactivated vaccines may still be administered safely [33]. Therefore, broadly postponing all vaccinations in these circumstances is generally unwarranted.

Rituximab, an anti-CD20 monoclonal antibody commonly administered before and after HCT, is associated with prolonged B-cell depletion. Current guidelines recommend delaying vaccination for at least six months following the last rituximab dose [33]. In contrast, low-dose corticosteroid therapy (<0.5 mg/kg prednisolone or equivalent) does not typically necessitate postponement of vaccines [5]. For patients receiving high-dose steroids or combination immunosuppressive regimens, vaccination timing should be assessed on a case-by-case basis, considering both clinical status and immune recovery [5].

Data remain limited regarding the effects of other immunosuppressive agents or the optimal timing of vaccinations following intravenous immunoglobulin (IVIG) administration [40,41]. These gaps in evidence contribute to variability in practice and underscore the need for more precise, evidence-based vaccination recommendations.

In conclusion, while patient compliance plays a role in revaccination delays, institutional and systemic factors—such as communication gaps and complex scheduling—represent primary barriers to effective vaccine delivery in HCT recipients. Addressing these challenges requires coordinated efforts among transplant teams, vaccination centers, and caregivers, supported by education, tracking systems, and individualized patient management strategies.

## 4. Chimeric Antigen Receptor T-Cell (CAR-T) Therapy and Infectious Risk: Implications for Immune Monitoring and Vaccination

CAR-T therapy is a novel immuno- and gene therapy approach involving the engineering of autologous or allogeneic T lymphocytes to express synthetic receptors that recognize specific cell-surface antigens, most commonly those expressed on malignant cells but not exclusively tumor-restricted [8,42]. These chimeric receptors combine an extracellular antigen-binding domain with intracellular signaling and co-stimulatory domains, enabling T cells to recognize antigen independently of MHC presentation and to mount a potent cytotoxic response. Currently, CAR-T therapy is primarily used for relapsed or refractory B-cell acute lymphoblastic leukemia (B-ALL) and large B-cell lymphoma, with CD19 as the most frequently targeted antigen. Ongoing research is expanding CAR-T applications to other hematologic malignancies, including multiple myeloma (e.g., BCMA-directed CAR-T) and acute myeloid leukemia, as well as selected solid tumors [42]. However, by targeting antigens that may also be expressed on normal cells, CAR-T therapy induces unique long-term immune deficits—such as B-cell aplasia and hypogammaglobulinemia in CD19-directed products—that profoundly affect vaccine responsiveness and necessitate tailored revaccination strategies.

Infections remain a significant complication associated with CAR-T cell therapy, with reported incidence rates ranging from 18% to 60%, particularly during the early post-infusion period (≤30 days) [8,43]. These early infections are often bacterial and correlate with neutropenia induced by lymphodepleting chemotherapy or treatment-related toxicities such as cytokine release syndrome (CRS). Viral infections predominate during the later post-infusion phase (days 30–90), primarily due to B-cell aplasia and hypogammaglobulinemia—hallmark consequences of CD19-directed CAR-T therapy. Frequently detected viral pathogens include respiratory viruses, cytomegalovirus (CMV), Epstein–Barr virus (EBV), and parvovirus B19. Although fungal infections are less common, they may occur in the context of prolonged neutropenia, corticosteroid therapy, or broad-spectrum antimicrobial use [8,43].

A retrospective study of 79 children with relapsed/refractory B-ALL reported an overall infection rate of 67.1% within 90 days post-CAR-T infusion, with bacterial infections predominating early and viral infections more frequent in later phases [44,45]. Risk factors for early infections, as identified by Wu et al., included high pre-infusion bone marrow disease burden, lymphopenia, post-infusion cytopenias, severe CRS or ICANS, corticosteroid and IL-6 receptor antagonist use, ICU admission, and elevated regulatory T-cell (Treg) levels. Treg expansion may simultaneously impair CAR-T efficacy and increase susceptibility to infections [43].

Immune reconstitution after CAR-T therapy is delayed, particularly concerning humoral recovery. In a study by Wang et al., CD8^+^ T cells recovered first (median 21 days), followed by NK cells (median 28 days), while CD4^+^ T-cell recovery was notably delayed. Universal B-cell aplasia occurred, with persistent immunoglobulin deficiencies, especially IgA, which remained suppressed beyond one year [46]. Similarly, a prospective pediatric cohort study by Deyà-Martínez et al. demonstrated ongoing B-cell aplasia up to 24 months post-infusion [47]. Immunoglobulin replacement therapy (IgRT) was initiated empirically, although only a minority of patients were completely agammaglobulinemic, suggesting possible persistence of IgA-producing plasma cells. These findings question the universal need for long-term IgRT and support a more individualized approach based on immunoglobulin profiling [48].

Even after quantitative B-cell recovery, functional deficits often persist, supporting the ongoing use of IgRT and highlighting the necessity for tailored vaccination schedules [49,50]. Walti et al. documented impaired humoral responses to vaccines in adult CAR-T recipients, although partial responses were noted in some, indicating residual B-cell or plasma cell function [50].

Vaccination strategies for CAR-T recipients should largely mirror those established for HCT patients, with adaptations based on immune status. Inactivated vaccines may be resumed approximately six months post-infusion in patients achieving remission, whereas live-attenuated vaccines should be deferred until at least 12 months post-treatment, contingent on documented immune recovery [49,50]. Priority vaccines include pneumococcal, influenza, and hepatitis B immunizations, particularly in regions with high endemicity or patient-specific risk factors. The recombinant zoster vaccine may be considered for seropositive patients. When feasible, influenza and SARS-CoV-2 vaccinations are recommended prior to CAR-T therapy. Post-CAR-T, mRNA-based SARS-CoV-2 vaccines can be administered from three months onward, with a complete primary series and booster doses advised [49,50,51]. Household contacts should also be vaccinated against influenza, measles, and varicella to provide indirect protection [44,45].

In patients previously undergoing HCT, vaccination planning should consider prior immunization responses and post-HCT vaccine status [44,45]. If protective titers were achieved after HCT and prior to CAR-T therapy, further vaccination may not be required. Conversely, incomplete vaccination or suboptimal responses necessitate revaccination following CAR-T therapy once immune recovery is confirmed. Verification of pneumococcal and hepatitis B immunity is crucial for patients preparing for CAR-T therapy following HCT [44,45].

A comprehensive, personalized approach—including immune monitoring, antimicrobial prophylaxis, IgRT, and tailored vaccination schedules—is essential to mitigate infectious risks and optimize long-term outcomes for pediatric patients undergoing CAR-T therapy.

## 5. Conclusions

Immunization is a cornerstone of survivorship care for pediatric patients following HCT and CAR-T therapy, both of whom remain highly vulnerable to vaccine-preventable diseases due to prolonged immunosuppression. In HCT recipients, immune deficits arise mainly from conditioning regimens and GVHD, resulting in variable but relatively predictable recovery timelines. By contrast, CAR-T therapy induces persistent B-cell aplasia and hypogammaglobulinemia, often leading to delayed or suboptimal vaccine responses. These fundamental differences demand tailored vaccination schedules, careful immune monitoring, and integrated infection-prevention strategies.

This review summarizes current recommendations from ECIL, the Centers for Disease Control and Prevention, the Infectious Diseases Society of America, the American Society for Blood and Marrow Transplantation, and the European Society for Blood and Marrow Transplantation, providing a structured framework for clinical practice. Nevertheless, adherence to these guidelines remains inconsistent, underscoring the need for coordinated, multidisciplinary implementation.

In addition, respiratory syncytial virus (RSV) has emerged as an important target for immunoprevention. Recently approved vaccines for older adults and maternal immunization, along with the long-acting monoclonal antibody nirsevimab for infants, highlight the rapidly evolving landscape of RSV protection [52,53,54,55]. Although data in pediatric HCT and CAR-T recipients remain limited, these strategies are highly relevant given the substantial morbidity and mortality associated with RSV in this population and are likely to be incorporated into future guideline updates.

Looking forward, research priorities include refining immunologic thresholds for the safe use of live vaccines, developing formulations that enhance immunogenicity in immunocompromised hosts, and applying immune profiling to personalize revaccination. As CAR-T therapy expands into broader indications and earlier treatment lines, understanding its unique immunological effects will be crucial to integrating vaccination into survivorship care. Ultimately, optimizing immunization in pediatric HCT and CAR-T recipients is essential to reduce infectious risk, improve quality of life, and sustain durable remission.

## Figures and Tables

**Table 1:** Recommended vaccines for HCT recipients’ children.

Type of Vaccine	Clinical Impact of the Disease	Recommendation	Features of Pediatric Population	Vaccine Efficacy
PCV	The risk of invasive pneumococcal disease (meningitis and pneumonia) is around 3.8–5.0 of 1000 transplant cases after autologous HCT and 8.2–9.0 of 1000 transplant cases after allogeneic HCT.	From 3 months after transplantation, three doses of PCV at 1-month intervals are recommended, followed by a dose of PPSV23 6 months later.	The administration of PPSV23 is recommended after the administration of three doses of PCV to broaden the spectrum of vaccination.	The response to three doses of PCV is 64–98%.
Hib	Hib can cause pneumonia, sinusitis, and bacteremia soon after transplantation.	From 3 months after transplantation, three doses of Hib vaccine at 1-month intervals are recommended. No preference on the type of vaccine (conjugated with tetanus-protein or diphtheria-protein). Alternatively, to decrease the overall number of vaccine doses, three doses of a combined diphtheria–tetanus–pertussis–Hib vaccine from 6 months after the transplantation.	NA	The response to Hib conjugate vaccines is 80–95%.
*Neisseria meningitidis*	The risk of invasive disease (meningitis) is higher after HCT. No clear data available in the literature.	From 6 months after transplantation, at least two doses of either a monovalent or tetravalent C vaccine and meningococcal B vaccine in accordance with country recommendations.	Children and adolescents are the main at-risk population.	The response to three doses of MCV-4 administered 12 months. After autologous HCT or 18 months after allogeneic transplantation is 100%. No data about MenB vaccine response.
Diphtheria and tetanus	Tetanus: The exposure to tetanus in the environment is a real risk for HCT patients. Diphtheria: There are very limited published data on Diphteritis in HCT. Ongoing vaccination is critical for immunity. Pertussis: There are very limited published data on pertussis in HCT.	From 6 months after transplantation, three doses of diphtheria–tetanus vaccine at 1–2-month intervals are recommended. Booster doses should be administered according to country recommendations. The ECIL group recommends considering the addition of pertussis toxoid to each dose of the diphtheria–tetanus vaccine.	DT vaccines should be preferred over Td vaccines.	The response to tetanus vaccine administered at 6–12 months after the transplantation is 85–100% after three doses. The response rate to diphtheria vaccine is 70–100% after three doses administered from 3 months after allogeneic HCT and 18 months to 10 years after autologous HCT No data about anti-pertussis vaccine response.
IIV	— One-third of HCT with confirmed influenza develop pneumonia. — 10% require mechanical ventilation — 6% died. — Other complications: encephalitis and myocarditis.	In the first year after HCT, two doses of IIV given one month apart are recommended to enhance immunogenicity, followed by a single annual dose at the start of each influenza season for as long as the patient remains immunocompromised.	Additional measures are also fundamental (e.g., respiratory isolation, rapid diagnostic tests in case of symptoms, and post-exposure antivirals). The intranasal influenza live-attenuated vaccine is contraindicated after HCT.	A systematic review and meta-analysis showed significantly lower odds of influenza-like illness after vaccination in transplant recipients compared with patients receiving placebo or no vaccination. Seroconversion and seroprotection are lower in transplant recipients compared with immunocompetent controls
HBV	Viral hepatitis can be life-threatening in patients with HCT recipients, because of the virus itself, or through a need to decrease the dose of chemotherapy.	Although most recent guidelines do not consider serostatus a decisive factor for revaccination, patients who are HBV-seronegative prior to transplantation, as well as those who were vaccinated before transplant but lose immunity within 6 months afterward, should be revaccinated according to national recommendations, typically with a three-dose series initiated 6–12 months post-transplant and administered at 0, 1, and 6 months, with post-vaccination serology used to confirm response.	Before transplant, patients who are negative for all HBV markers and are transplanted with a graft from an anti-HBc-positive donor should be vaccinated if possible and could additionally receive anti-HBV immunoglobulins. Patients infected with HBV before HCT (HBsAg-negative and anti-HBc-positive) should be assessed regularly for anti-HBs antibody titers and should be vaccinated if they have unprotective titers. If anti-HBs titers are <10 mIU/mL 1–2 months after the initial series of three vaccine doses, an additional series of three doses should be considered. Children should receive a standard pediatric dose (10 µg) of vaccine, and adolescents should receive 20 µg of the vaccine according to the summary of product characteristics of each vaccine.	The seroconversion rate was nearly 64%.
HPV	Genital HPV disease is a significant late complication of alloHCT, occurring in one-third of women. In long-term survivors, second neoplasias are a significant complication after alloHCT. Cervix cancer is one of the most frequent. Squamous cell cancers, the commonest post-transplant solid tumors, are associated with HPV infection.	From 6 to 12 months after transplantation, recommendations for the general population in each country should be followed.	NA	The seroconversion rate was nearly 100%.
IPV	NA Maintaining high vaccination coverage in all population groups remains an essential tool for keeping Europe polio-free.	From 6 to 12 months after transplantation, three doses of PV are recommended to be administered at 1–2-month intervals; booster doses should be administered according to country recommendations.	NA	The response to three doses of the vaccine administered 6 months after the transplant is 80–100% and long-lasting.
Measles–mumps–rubella	Measles: Severe and also fatal measles infections (pneumonia, encephalitis) have been reported in HCT recipients. Rubella: The main indication for rubella vaccination is prevention of congenital rubella in fertile women. Mumps: There are no reports of severe mumps occurring in HCT recipients.	Starting from 24 months post-transplant, live vaccines should be considered only in seronegative patients who are free of GVHD, off immunosuppressive therapy, without disease relapse, and with no recent administration of immunoglobulins.	Seronegative patients for measles should receive one dose of MMR; HCT recipients who are women, seronegative for rubella, and of childbearing potential should receive one dose of MMR; in case of a measles outbreak, MMR vaccination could be considered 12 months after transplantation in patients with low-grade immunosuppression	The response rate is 65–100% for measles, 50–87% for mumps, and 75–100% for rubella.
VZV	VZV infections could be life-threatening after HCT. The risks are primary varicella infection (chickenpox) in seronegative patients and shingles and postherpetic neuralgia in seropositive patients.	From 24 months after transplantations, only in seronegative patients with no GvHD, no immunosuppressants, no relapse, and no recent administration of immunoglobulins.	Some patients developed vaccine-related varicella.	The response rate is around 65%, without a clear benefit of a second dose.

Adapted from references [5,7]. DT, diphtheria–tetanus vaccine; ECIL, European Conference on Infections in Leukaemia guidelines; GvHD, graft-versus-host disease; HBV, hepatitis B virus; HCT, hematopoietic cell transplantation; Hib, *Haemophilus influenzae* type b; HPV, human papillomavirus; IPV, inactivated poliovirus vaccine; MCV, meningococcal conjugate vaccine; menB, meningococcal B vaccine; MMR, measles, mumps, rubella; NA, not applicable; PCV, pneumococcal conjugate vaccine; PPSV23, 23-valent pneumococcal polysaccharide vaccine; Td, tetanus–diphtheria vaccine.; VZV, varicella zoster virus.

## Data Availability

Not applicable.

## References

[1] Wohlschlaeger A., Levy E., Khan R.N., Heimall J., Fisher B.T., Metjian T.A., Elgarten C.W., Freedman J.L. (2023). A Retrospective Review of Revaccination Patterns in Pediatric Hematopoietic Stem Cell Transplantation Recipients. J. Pediatr. Hematol. Oncol. Nurs..

[2] Miller P.D.E., de Silva T.I., Skinner R., Gilleece M., Peniket A., Hamblin A., Greenfield D., Anthias C., Peggs K., Madrigal A. (2017). Routine vaccination practice after adult and paediatric allogeneic haematopoietic stem cell transplant: A survey of UK NHS programmes. Bone Marrow Transplant..

[3] Kumar D., Humar A., Plevneshi A., Siegal D., Franke N., Green K., McGeer A., Toronto Invasive Bacterial Diseases Network (2008). Invasive pneumococcal disease in adult hematopoietic stem cell transplant recipients: A decade of prospective population-based surveillance. Bone Marrow Transplant..

[4] Ge Y.L., Zhai X.W., Zhu Y.F., Wang X.S., Xia A.M., Li Y.F., Zeng M. (2017). Measles Outbreak in Pediatric Hematology and Oncology Patients in Shanghai, 2015. Chin. Med. J..

[5] Miller P., Patel S.R., Skinner R., Dignan F., Richter A., Jeffery K., Khan A., Heath P.T., Clark A., Orchard K. (2023). Joint consensus statement on the vaccination of adult and paediatric haematopoietic stem cell transplant recipients: Prepared on behalf of the British society of blood and marrow transplantation and cellular therapy (BSBMTCT), the Children’s cancer and Leukaemia Group (CCLG), and British Infection Association (BIA). J. Infect..

[6] Danino D., Stanek J.R., Rangarajan H., Ardura M.I. (2021). Hospitalizations for vaccine-preventable infections among pediatric hematopoietic cell transplantation recipients in the first 5 years after transplantation. Bone Marrow Transplant..

[7] Neemann K.A., Sato A.I. (2023). Vaccinations in children with hematologic malignancies and those receiving hematopoietic stem cell transplants or cellular therapies. Transpl. Infect. Dis..

[8] Kansagra A.J., Frey N.V., Bar M., Laetsch T.W., Carpenter P.A., Savani B.N., Heslop H.E., Bollard C.M., Komanduri K.V., Gastineau D.A. (2019). Clinical Utilization of Chimeric Antigen Receptor T Cells in B Cell Acute Lymphoblastic Leukemia: An Expert Opinion from the European Society for Blood and Marrow Transplantation and the American Society for Blood and Marrow Transplantation. Biol. Blood Marrow Transplant..

[9] Van Veen K.E., Brouwer M.C., van der Ende A., van de Beek D. (2016). Bacterial meningitis in hematopoietic stem cell transplant recipients: A population-based prospective study. Bone Marrow Transplant..

[10] Shigayeva A., Rudnick W., Green K., Chen D.K., Demczuk W., Gold W.L., Johnstone J., Kitai I., Krajden S., Lovinsky R. (2016). Invasive Pneumococcal Disease Among Immunocompromised Persons: Implications for Vaccination Programs. Clin. Infect. Dis..

[11] Cordonnier C., Bernaudin J.F., Bierling P., Huet Y., Vernant J.P. (1986). Pulmonary complications occurring after allogeneic bone marrow transplantation. A study of 130 consecutive transplanted patients. Cancer.

[12] Ljungman P., de la Camara R., Perez-Bercoff L., Abecasis M., Nieto Campuzano J.B., Cannata-Ortiz M.J., Cordonnier C., Einsele H., Gonzalez-Vicent M., Espigado I. (2011). Outcome of pandemic H1N1 infections in hematopoietic stem cell transplant recipients. Haematologica.

[13] Beck C.R., McKenzie B.C., Hashim A.B., Harris R.C., Nguyen-Van-Tam J.S., University of Nottingham Influenza and the ImmunoCompromised (UNIIC) Study Group (2012). Influenza vaccination for immunocompromised patients: Systematic review and meta-analysis by aetiology. J. Infect. Dis..

[14] Mallet V., van Bömmel F., Doerig C., Pischke S., Hermine O., Locasciulli A., Cordonnier C., Berg T., Moradpour D., Wedemeyer H. (2016). Management of viral hepatitis in patients with haematological malignancy and in patients undergoing haemopoietic stem cell transplantation: Recommendations of the 5th European Conference on Infections in Leukaemia (ECIL-5). Lancet Infect. Dis..

[15] Shanis D., Anandi P., Grant C., Bachi A., Vyas N., Merideth M.A., Pophali P.A., Koklanaris E., Ito S., Savani B.N. (2018). Risks factors and timing of genital human papillomavirus (HPV) infection in female stem cell transplant survivors: A longitudinal study. Bone Marrow Transplant..

[16] Haynes A.S., Curtis D.J., Campbell K., Giller R.H., Quinones R.R., Verneris M.R., Abzug M.J. (2021). An Immune Recovery-Based Revaccination Protocol for Pediatric Hematopoietic Stem Cell Transplant Recipients: Revaccination Outcomes Following Pediatric HSCT. Transplant. Cell Ther..

[17] Greco R., Ciceri F., Noviello M., Bondanza A., Vago L., Oliveira G., Peccatori J., Cieri N., Ruggeri A., Koehl U. (2018). Immune monitoring in allogeneic hematopoietic stem cell transplant recipients: A survey from the EBMT-CTIWP. Bone Marrow Transplant..

[18] Wiegering V., Eyrich M., Winkler B., Schlegel P.G. (2019). Comparison of Immune Reconstitution After Allogeneic Versus Autologous Stem Cell Transplantation in 182 Pediatric Recipients. J. Pediatr. Hematol. Oncol..

[19] Cecinati V., Principi N., Brescia L., Esposito S. (2014). Antibiotic prophylaxis in children with cancer or who have undergone hematopoietic cell transplantation. Eur. J. Clin. Microbiol. Infect. Dis..

[20] Olkinuora H., von Willebrand E., Kantele J.M., Vainio O., Talvensaari K., Saarinen-Pihkala U., Siitonen S., Vettenranta K. (2011). The impact of early viral infections and graft-versus-host disease on immune reconstitution following paediatric stem cell transplantation. Scand. J. Immunol..

[21] Forlenza C.J., Small T.N. (2013). Live (Vaccines) from New York. Bone Marrow Transplant..

[22] Small T.N., Zelenetz A.D., Noy A., Rice R.D., Trippett T.M., Abrey J.D., Portlock C.S., McCullagh E.J., Vanak J.M., Mulligan A.M. (2009). Pertussis immunity and response to tetanus-reduced diphtheria-reduced pertussis vaccine (Tdap) after autologous peripheral blood stem cell transplantation. Biol. Blood Marrow Transplant..

[23] Ljungman P., Cordonnier C., Einsele H., Englund J., Machado C.M., Storek J., Small T. (2009). Vaccination of hematopoietic cell transplant recipients. Bone Marrow Transplant..

[24] Amarin J.Z., Dulek D.E., Simmons J., Hayek H., Chappell J.D., Nochowicz C.H., Kitko C.L., Schuster J.E., Muñoz F.M., Bocchini C.E. (2024). Immunophenotypic predictors of influenza vaccine immunogenicity in pediatric hematopoietic cell transplant recipients. Blood Adv..

[25] Al-Antary E., Henry M., Spruit J., Yankelevich M., Chu R., Ravindranath Y., Savaşan S. (2021). Patterns and correlates of preserved humoral immunity to vaccines in children following allogeneic hematopoietic stem cell transplantation. Pediatr. Transplant..

[26] Ozboru Askan O., Ozden T.A., Karasu Tezcan G., Keskindemirci G., Bakir A., Tugcu D., Pekun F., Yesilipek A., Gokcay E.G. (2023). Vaccine Adherence and Postvaccination Serological Status of Pediatric Allogeneic Hematopoietic Stem Cell Transplant Recipients: A Single-center Experience. J. Pediatr. Hematol. Oncol..

[27] Sattler C., Hoffmann P., Herzberg P.Y., Weber D., Holler B., Fehn U., Plentz A., Beckhove P., Winkler J., Edinger M. (2021). Primary vaccination in adult patients after allogeneic hematopoietic stem cell transplantation—A single center retrospective efficacy analysis. Vaccine.

[28] Lee E.S., Kim S.K., Han S.B., Lee J.W., Chung N.G., Cho B., Jeong D.C., Kang J.H. (2023). Serologic status and vaccine response against hepatitis B virus after allogeneic hematopoietic cell transplantation in pediatric patients. Asian Pac. J. Allergy Immunol..

[29] Olkinuora H., Käyhty H., Davidkin I., Roivainen M., Ölander R.-M., Kantele J.M., Siitonen S., Vettenranta K. (2012). Immunity after (re)vaccination of paediatric patients following haematopoietic stem cell transplantation. Acta Paediatr..

[30] Kondolot M., Yilmaz E., Erdog Sahin N., Ozcan A., Kaynar L., Unal E., Karakukcu M. (2023). Antibody Response against Vaccine Antigens in Children after TCRαβ-Depleted Haploidentical Stem Cell Transplantation: Is It Similar to That in Recipients with Fully Matched Donors?. Transplant. Cell Ther..

[31] Pettke A., Jocham S., Wiener A., Löcken A., Groenefeld J., Ahlmann M., Groll A.H. (2017). Vaccination against influenza at a European pediatric cancer center: Immunization rates and attitudes among staff, patients, and their families. Support. Care Cancer.

[32] Freedman J.L., Reilly A.F., Powell S.C., Bailey L.C. (2015). Quality improvement initiative to increase influenza vaccination in pediatric cancer patients. Pediatrics.

[33] Cordonnier C., Einarsdottir S., Cesaro S., Di Blasi R., Mikulska M., Rieger C., de Lavallade H., Gallo G., Lehrnbecher T., Engelhard D. (2019). Vaccination of haemopoietic stem cell transplant recipients: Guidelines of the 2017 European Conference on Infections in Leukaemia (ECIL 7). Lancet Infect. Dis..

[34] Seale H., Leask J., MacIntyre C.R. (2010). Attitudes amongst Australian hospital healthcare workers towards seasonal influenza and vaccination. Influenza Other Respir. Viruses.

[35] Astray-Mochales J., López de Andres A., Hernandez-Barrera V., Rodríguez-Rieiro C., Carrasco Garrido P., Esteban-Vasallo M.D., Domínguez-Berjón M.F., Jimenez-Trujillo I., Jiménez-García R. (2016). Influenza vaccination coverages among high risk subjects and health care workers in Spain. Results of two consecutive National Health Surveys (2011–2014). Vaccine.

[36] Esposito S., Tremolati E., Bellasio M., Chiarelli G., Marchisio P., Tiso B., Mosca F., Pardi G., Principi N. (2007). Attitudes and knowledge regarding influenza vaccination among hospital health workers car- ing for women and children. Vaccine.

[37] Silva P.M.D., Silva É.M.D., Simioni A.J., Souza M.P., Colturato V.A.R., Machado C.M. (2017). Difficulties in the revaccination program of hematopoietic stem cell transplantation recipients. Rev. Inst. Med. Trop. Sao Paulo.

[38] Nelson J.C., Bittner R.C., Bounds L., Zhao S., Baggs J., Donahue J.G., Hambidge S.J., Jacobsen S.J., Klein N.P., Naleway A.L. (2009). Compliance with multiple-dose vaccine schedules among older children, adolescents, and adults: Results from a vaccine safety datalink study. Am. J. Public Health.

[39] Buchbinder D., Brazauskas R., Bo-Subait K., Ballen K., Parsons S., John T., Hahn T., Sharma A., Steinberg A., D’SOuza A. (2020). Predictors of Loss to Follow-Up Among Pediatric and Adult Hematopoietic Cell Transplantation Survivors: A Report from the Center for International Blood and Marrow Transplant Research. Biol. Blood Marrow Transplant..

[40] Hudspeth M.P., Hill T.N., Lewis J.A., Van Meter E., Ragucci D. (2010). Post-hematopoietic stem cell transplant immunization practices in the Pediatric Blood and Marrow Transplant Consortium. Pediatr. Blood Cancer.

[41] Ariza-Heredia E.J., Gulbis A.M., Stolar K.R., Kebriaei P., Shah D.P., McConn K.K., Champlin R.E., Chemaly R.F. (2014). Vaccination guidelines after hematopoietic stem cell transplantation: Practitioners’ knowledge, attitudes, and gap between guidelines and clinical practice. Transpl. Infect. Dis..

[42] Yakoub-Agha I., Chabannon C., Bader P., Basak G.W., Bonig H., Ciceri F., Corbacioglu S., Duarte R.F., Einsele H., Hudecek M. (2020). Management of adults and children undergoing chimeric antigen receptor T-cell therapy: Best practice recommendations of the European Society for Blood and Marrow Transplantation (EBMT) and the Joint Accreditation Committee of ISCT and EBMT (JACIE). Haematologica.

[43] Wu X., Cao Z., Chen Z., Wang Y., He H., Xiao P., Hu S., Lu J., Li B. (2024). Infectious complications in pediatric patients undergoing CD19+CD22+ chimeric antigen receptor T-cell therapy for relapsed/refractory B-lymphoblastic leukemia. Clin. Exp. Med..

[44] Los-Arcos I., Iacoboni G., Aguilar-Guisado M., Alsina-Manrique L., de Heredia C.D., Fortuny-Guasch C., García-Cadenas I., García-Vidal C., González-Vicent M., Hernani R. (2021). Recommendations for screening, monitoring, prevention, and prophylaxis of infections in adult and pediatric patients receiving CAR T-cell therapy: A position paper. Infection.

[45] Shahid Z., Jain T., Dioverti V., Pennisi M., Mikkilineni L., Thiruvengadam S.K., Shah N.N., Dadwal S., Papanicolaou G., Hamadani M. (2024). Best Practice Considerations by The American Society of Transplant and Cellular Therapy: Infection Prevention and Management After Chimeric Antigen Receptor T Cell Therapy for Hematological Malignancies. Transplant. Cell Ther..

[46] Wang Y., Li H., Song X., Qi K., Cheng H., Cao J., Shi M., Yan Z., Jing G., Pan B. (2021). Kinetics of immune reconstitution after anti-CD19 chimeric antigen receptor T cell therapy in relapsed or refractory acute lymphoblastic leukemia patients. Int. J. Lab. Hematol..

[47] Deyà-Martínez A., Alonso-Saladrigues A., García A.P., Faura A., Torrebadell M., Vlagea A., Català A., Esteve-Solé A., Juan M., Rives S. (2021). Kinetics of humoral deficiency in CART19-treated children and young adults with acute lymphoblastic leukaemia. Bone Marrow Transplant..

[48] Walti C.S., Krantz E.M., Maalouf J., Boonyaratanakornkit J., Keane-Candib J., Joncas-Schronce L., Stevens-Ayers T., Dasgupta S., Taylor J.J., Hirayama A.V. (2021). Antibodies against vaccine-preventable infections after CAR-T cell therapy for B cell malignancies. JCI Insight.

[49] Gonzalez M.A., Boonyaratanakornkit J., Bhatti A., Keane-Candib J., Huang M.-L., Campbell V., Goecker E., Ibrahimi S., Hecht J., McClurkan C. (2022). Comparison of humoral and T-cell response after SarsCov-2 vaccination among patients before and after chimeric antigen receptor-modified T cell (CAR-T cell) therapy. Transplant. Cell Ther..

[50] Walti C.S., Loes A.N., Shuey K., Krantz E.M., Boonyaratanakornkit J., Keane-Candib J., Loeffelholz T., Wolf C.R., Taylor J.J., Gardner R.A. (2021). Humoral immunogenicity of the seasonal influenza vaccine before and after CAR-T-cell therapy: A prospective observational study. J. Immunother. Cancer.

[51] Khawaja F., Papanicolaou G., Dadwal S., Pergam S.A., Wingard J.R., El Boghdadly Z., Abidi M.Z., Waghmare A., Shahid Z., Michaels L. (2023). Frequently asked questions on Coronavirus Disease 2019 vaccination for hematopoietic cell transplantation and chimeric antigen receptor T-cell recipients from the american society for transplantation and cellular therapy and the American Society of Hematology. Transplant. Cell Ther..

[52] Riccò M., Abu-Raya B., Icardi G., Spoulou V., Greenberg D., Pecurariu O.F., Hung I.F., Osterhaus A., Sambri V., Esposito S. (2024). Respiratory Syncytial Virus: A WAidid Consensus Document on New Preventive Options. Vaccines.

[53] Principi N., Perrone S., Esposito S. (2025). Challenges and Limitations of Current RSV Prevention Strategies in Infants and Young Children: A Narrative Review. Vaccines.

[54] Esposito S., Abu Raya B., Baraldi E., Flanagan K., Martinon Torres F., Tsolia M., Zielen S. (2022). RSV Prevention in All Infants: Which Is the Most Preferable Strategy?. Front. Immunol..

[55] Esposito S., Abu-Raya B., Bonanni P., Cahn-Sellem F., Flanagan K.L., Martinon Torres F., Mejias A., Nadel S., Safadi M.A.P., Simon A. (2021). Coadministration of Anti-Viral Monoclonal Antibodies with Routine Pediatric Vaccines and Implications for Nirsevimab Use: A White Paper. Front. Immunol..

